# Assessment of Genetic Differentiation and Linkage Disequilibrium in *Solanum pimpinellifolium* Using Genome-Wide High-Density SNP Markers

**DOI:** 10.1534/g3.118.200862

**Published:** 2019-03-11

**Authors:** Ya-Ping Lin, Chu-Yin Liu, Kai-Yi Chen

**Affiliations:** Department of Agronomy, National Taiwan University, Taipei, Taiwan, 10617

**Keywords:** *Solanum pimpinellifolium*, genetic differentiation, linkage disequilibrium, RADseq

## Abstract

To mine new favorable alleles for tomato breeding, we investigated the feasibility of utilizing *Solanum pimpinellifolium* as a diverse panel of genome-wide association study through the restriction site-associated DNA sequencing technique. Previous attempts to conduct genome-wide association studies using *S. pimpinellifolium* were impeded by an inability to correct for population stratification and by lack of high-density markers to address the issue of rapid linkage disequilibrium decay. In the current study, a set of 24,330 SNPs was identified using 99 *S. pimpinellifolium* accessions from the Tomato Genetic Resource Center. Approximately 84% of *Pst*I site-associated DNA sequencing regions were located in the euchromatic regions, resulting in the tagging of most SNPs on or near genes. Our genotypic data suggested that *S. pimpinellifolium* were divided into three single-ancestry subpopulations and four mixed-ancestry subpopulations. Additionally, our SNP genotypic data consistently confirmed the genetic differentiation, achieving a relatively reliable correction of population stratification. Previous studies utilized the 8K tomato SNP array, SolCAP, to investigate the genetic variation of *S. pimpinellifolium* and we performed a meta-analysis of these genotypes. The result suggested SolCAP array was less appropriate to profile the genetic differentiation of *S. pimpinellifolium* when more accessions were involved because the samples belonging to the same accession demonstrated different genome patterns. Moreover, as expected, rapid linkage disequilibrium decay was observed in *S. pimpinellifolium*, especially in euchromatic regions. Approximately two-thirds of the flanking SNP markers did not display linkage disequilibrium based on *r^2^* = 0.1. However, the 18-Kb linkage disequilibrium decay indeed reveals the potential of single-gene resolution in GWAS when markers are saturated.

The wild tomato species *Solanum pimpinellifolium* is a native perennial shrub in Ecuador and Peru, ranging along the western Andean slopes to the coastal regions. It is believed that *S. pimpinellifolium* originated in northern Peru and then diversified into several subpopulations after it migrated to Ecuador and southern Peru (Rick *et al.* 1977; Zuriaga *et al.* 2009; Blanca *et al.* 2012, 2015). These regions present gradient temperature and precipitation changes from Ecuador toward southern Peru: western Ecuador is equatorial winter dry; northern Peru is hot, arid desert; southern Peru is cold, barren desert (Kottek *et al.* 2006; Moyle 2008; Zuriaga *et al.* 2009). Previous studies showed high genetic variation and high outcrossing rate of the accessions in northern Peru and revealed genetic differentiation between the accessions collected in Ecuador and those in Peru (Rick *et al.* 1977; Caicedo and Schaal 2004; Zuriaga *et al.* 2009; Rao *et al.* 2012). Recently, with the aid of SolCAP genotyping array, *S. pimpinellifolium* was divided into three subpopulations: one in northern Ecuador, one in the mountains of Ecuador extending to the north of Peru, and one in Peru (Blanca *et al.* 2012, 2015). Because geographic distributions of distinct *S. pimpinellifolium* subpopulations also aligned from north to south, the genetic distances between subpopulations were thought to correlate with climatic differences (Zuriaga *et al.* 2009; Blanca *et al.* 2012, 2015).

*S. pimpinellifolium* is an attractive resource for tomato breeding because it can freely cross with cultivated tomatoes and introduces novel alleles into the limited gene pool of cultivated tomatoes (Tanksley and Mccouch 1997; Spooner *et al.* 2005; Moyle 2008). *S. pimpinellifolium* has been used as a genetic resource for disease resistance and fruit quality traits in tomato breeding (Grandillo *et al.* 2011; Víquez-Zamora *et al.* 2014; Capel *et al.* 2015). A core collection of *S. pimpinellifolium* was developed at World Vegetable Center for preservation and utilization (Rao *et al.* 2012). This core collection has been used to mine novel alleles of salt tolerance via the candidate gene approach (Rao *et al.* 2015).

A comprehensive way to mine new favorable alleles in wild species is to conduct a genome-wide association study (GWAS). GWAS utilizes linkage disequilibrium (LD), the non-random association between marker alleles and alleles conferring targeted phenotypes in a given collection of germplasm, to map quantitative trait loci (QTL) (Soto-Cerda and Cloutier 2012). In comparison with the traditional genetic mapping method using progenies derived from a bi-parental cross, GWAS usually brings higher mapping resolution because of more detectable recombinant events in a collection of germplasm. Previous studies revealed the average ranges of LD decay in different collections of cultivated tomatoes varied from 6.1 to 12.5 cM based on *r^2^* = 0.2 and 13.4 cM based on *r^2^* = 0.1 (Sim *et al.* 2012a; Blanca *et al.* 2015). Because *S. pimpinellifolium* presents greater genetic variation than cultivated tomatoes, the range of LD decay is expectedly smaller in *S. pimpinellifolium* populations (1.7 cM based on *r^2^* = 0.1) (Blanca *et al.* 2012, 2015; Ranc *et al.* 2012; Bauchet *et al.* 2017). However, the 8K SolCAP genotyping array and additional 6K CBSG genotyping array did not achieve full LD coverage across all chromosomal regions for *S. pimpinellifolium* accessions (Sim *et al.* 2012a, 2012b; Bauchet *et al.* 2017). The restriction site-associated DNA sequencing (RADseq) technique may provide an inexpensive solution to address this challenge (Davey and Blaxter 2010).

The RADseq technique limits sequencing resources at the vicinity of restriction enzyme cutting sites and therefore provides flexibility of experimental design regarding the trade-off between cost-effectiveness and marker densities (Chen *et al.* 2014; Bhakta *et al.* 2015). *Pst*I is a methylation-sensitive restriction enzyme and recognizes the sequences “CTGCAG” (Dobritsa and Dobritsa 1980). A study of the genome-wide methylation pattern in tomato leaves and immature fruits revealed that the gene-rich euchromatic regions at the distal ends of chromosomes were characterized as the regions with low levels of cytosine methylation at the “CG”, “CTG”, and “CAG” sequences and the pericentromeric heterochromatin regions were the regions with high levels of cytosine methylation (Zhong *et al.* 2013). DNA markers would be found mainly in the gene-rich euchromatic regions and sparsely in the heavily methylated heterochromatic regions when extracted genomic DNAs come from young tomato leaves and are digested with *Pst*I following the RADseq protocol. Because the euchromatin usually has a higher frequency of genetic recombination than the heterochromatin, this RADseq experimental design based on *Pst*I digestion could increase marker density in the chromosomal region with higher frequencies of genetic recombination and decrease marker density in the chromosomal region with lower frequencies of genetic recombination. This RADseq design may fulfill the demand of the high-density markers in GWAS using a *S. pimpinellifolium* collection.

The objective of the current study was first to develop genome-wide high-density SNP markers for a subset of *S. pimpinellifolium* collections from the Tomato Genetic Resource Center (TGRC) through the RADseq approach. Second, the population differentiation was examined by different methods to ensure a stable estimation. Moreover, a meta-analysis of SolCAP array was performed to infer the population differentiation in a scenario involving in more accessions. Third, the LD decay was assessed to estimate the required marker number and potential resolution in GWAS.

## Materials and Methods

### Plant materials

All plant materials and their information were obtained from TGRC (Table S1; http://tgrc.ucdavis.edu/). A total of 12 accessions from Ecuador and 87 accessions from Peru were utilized in this study. According to their mating types, 43 accessions were facultative self-compatible (FSC), and 56 accessions were autogamous self-compatible (ASC). Seeds were propagated by self-pollination for two generations using the method of single-seed descent in a greenhouse. Young leaves collected from plants of these single-seed descendent seeds were used for DNA extraction.

### RAD sequencing

Total genomic DNA was extracted from young leaves using a modified CTAB method (Fulton *et al.* 1995) and purified with a DNeasy Blood & Tissue Kit (QIAGEN, Venlo, Netherland) following the manufacturer’s instructions. We chose *Pst*I to select the sequencing regions because *Pst*I is a methylation-sensitive restriction enzyme and it may cut more frequently in euchromatin regions than heterochromatin regions (Dobritsa and Dobritsa 1980). *Pst*I-digested DNA libraries were prepared following the protocol of Etter *et al.* (Etter *et al.* 2011). Four RADseq libraries were constructed, and each was sequenced in one lane of an Illumina HiSeq2000 flow cell (100 bp single-end reads) (Illumina Inc., San Diego, CA, USA). All the sequences of RADseq were submitted to the NCBI SRA database, and the BioProject Number is PRJNA358110.

### SNP calling

Reads were analyzed with Stacks version 1.37 (Catchen *et al.* 2013) and with CLC Genomics Workbench software version 6.5.1 (QIAGEN, Venlo, Netherlands). First, the *process_radtags* command in Stacks filtered out low-quality reads with Q scores less than 20. The remaining reads were mapped to the tomato reference genome SL2.50 (Fernandez-Pozo *et al.* 2015) using the “Map Reads to Reference” tool in the CLC Genomics Workbench software. Considering that genetic variation between the tomato reference genome *S. lycopersicum* and *S. pimpinellifolium* is larger than genetic variation within *S. lycopersicum*, mapping parameters were set as 0.5 for the length fraction and 0.9 for the similarity fraction. The reads of the same individual in different lanes were merged. In the subsequent analyses using Stacks, the *ref_map.pl* command set the parameter –m (minimum read depth to create a stack) as 10, and the *populations* command set the parameter –p (minimum number of populations a locus must be present) as 75. SNPs with a minor allele frequency of less than 0.05 were further excluded, and a set of 24,330 SNP markers was obtained. This set of 24,330 SNP markers was utilized for the analyses of genetic variation, LD, *F_st_* and AMOVA. Another SNP set without ‘redundant SNP markers’ was used to conduct the principal component analysis (PCA) and ADMIXTURE because these two matrices are expected to correct the structure in GWAS. To remove ‘redundant SNP markers’, we defined a sequencing unit as a sequencing region surrounding a *Pst*I site, usually 186 bp long, which has at least one SNP with a minor allele frequency greater than 0.05 in the *S. pimpinellifolium* population. If more than one SNPs are located in a sequencing unit and they are in complete LD (*r^2^* = 1), only the first SNP is kept. This process resulted in a total of 19,993 SNP markers. ITAG2.4 gene model from SGN was used as the reference gene annotation.

### Population differentiation

PCA was performed in TASSEL5.0 (Bradbury *et al.* 2007). ADMIXTURE was completed following by the manual; the best K was determined following the procedure of cross-validation in the manual (Alexander *et al.* 2009). Pairwise *F_st_* (Weir and Cockerham 1984) and analysis of molecular variance (AMOVA) (Excoffier *et al.* 1992) were conducted in the R package StAMPP (Pembleton *et al.* 2013). Details of the analysis of isolation by distance and the meta-analysis of *S. pimpinellifolium* SolCAP genotyping data are described in the supplements, File S1, and File S2, respectively.

### Estimation of genetic variation and LD

Genetic variation within overall accessions and within each of the seven groups was assessed based on observed heterozygosity and the within-population gene diversity (expected heterozygosity) using the R package hierfstat (Goudet and Jombart 2015). Pairwise *r^2^* values between SNP markers were calculated to assess overall extent of LD via plink1.9 within a 1-Mb window (Gaunt *et al.* 2007) and fit by non-linear regression (Remington *et al.* 2001). The baseline of the *r^2^* value was set at 0.1 (Bauchet *et al.* 2017). The local LD along each chromosome was assessed as follows: for each pair of consecutive sequencing units (defined in the section of SNP calling), the average *r^2^* was calculated between two SNPs in different sequencing units and plotted along the left *Pst*I cutting site based on the physical position. The heterochromatin regions were marked according to the genetic map of EXPIM 2012 and the physical map of the tomato reference genome (Sim *et al.* 2012b).

### Data availability

Data available: NCBI SRA BioProject Number PRJNA358110. The authors affirm that all data necessary for confirming the conclusions of this article are represented fully within the article and its tables and figures. Supplemental material available at Figshare: https://doi.org/10.25387/g3.7730369.

## Results

### Identification of 24,330 SNPs From PstI-digested DNA libraries

A total of 655,973,270 short DNA reads were obtained from four lanes of the Illumina HiSeq2000 flow cell and were divided into 99 parts according to barcode sequences. Each part was derived from the DNA of a *S. pimpinellifolium* accession and contained at least 3.7 million DNA reads, except for LA2647 (Table S1). Among the 82,814 *Pst*I sites in the tomato reference sequence SL2.50, only 23,988 *Pst*I sites were covered by the sequenced DNA reads (Table S2). The sequenced regions included 0.54% of the SL2.50 reference sequences and 12,790 annotated genes (Table 1). Interestingly, approximately 84% of the sequenced *Pst*I sites were located in the euchromatic regions (Table S2). Besides, the proportion of sequenced genes in euchromatin (43.13%) were about twice as that in heterochromatin (19.75%) (Table S2).

**Table 1:** Summary of the markers developed with the RAD sequencing strategy and the sequenced genes as well

Chr.	SNPs	Genes in sequenced regions	Genes with SNPs	SNPs in gene regions
0	147	62	25	57
1	3,222	1,742	1,029	2,374
2	2,401	1,400	803	1,661
3	2,522	1,389	812	1,779
4	2,121	1,054	611	1,328
5	1,680	783	437	1,049
6	2,179	1,195	673	1,422
7	1,756	902	535	1,174
8	1,929	952	599	1,304
9	1,670	877	507	1,192
10	1,616	812	444	954
11	1,563	834	466	1,054
12	1,524	788	440	1,017
**Total**	**24,330**	**12,790**	**7,381**	**16,365**

Two criteria were set to ensure the accuracy of SNP calling and genotype calling: one was that the read depth aligning to the reference sequence was equal to or greater than 10, and the other was that at least 75% of the accessions showed genotypes associated with a defined SNP marker. A total of 67,804 SNPs were identified in the sequenced regions of 99 *S. pimpinellifolium* accessions, and 24,330 of them had the minor allele frequency higher than 0.05. In the genotypic dataset of the 24,330 SNP markers (Table S3), the missing proportion of each accession ranged from 0.72 to 15.92%, except for LA2647 of which the value was 65.68% due to a low number of sequencing reads (Table S1). Regarding the location of these 24,330 SNPs, 16,365 SNPs were found in 7,383 annotated genes (Table 1), and the remaining SNPs were in the intergenic regions. Concerning the proportion of sequenced *Pst*I sites that contained SNPs, there is no significant difference between those sites in euchromatin (68.85%) and those in heterochromatin (60.59%) (Table S2). Meanwhile, the genotypic data of the LA0411 accession was dropped because the observed heterozygosity of LA0411 was inconsistent with its mating type (Table S1).

### A similar distribution between genes and SNPs was identified in the vicinity of PstI cutting site throughout the genome

**Figure 1:**
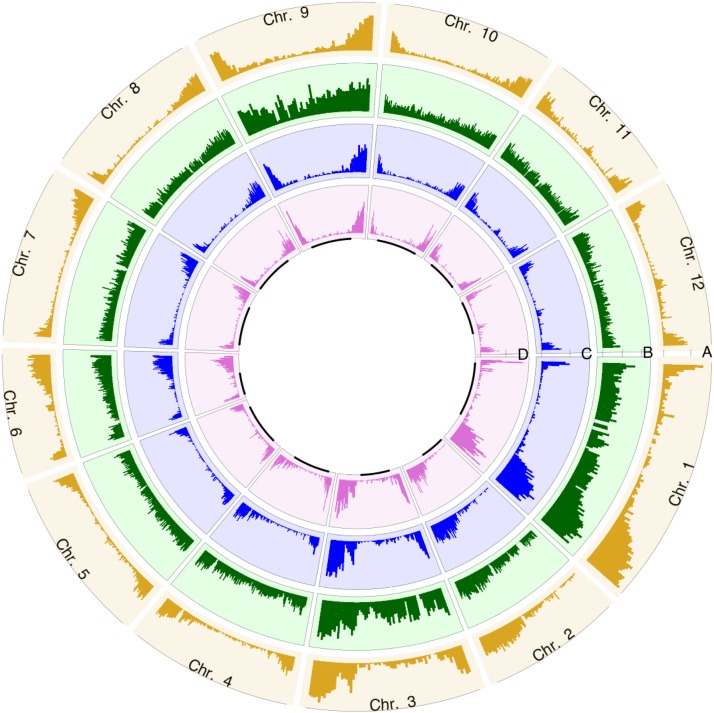
The distributions of ITAG2.4 gene model, *Pst*I cutting sites and SNPs throughout the genome. Each section indicates one chromosome, with labeling on the circumference. Circles A, B, C, and D indicate the distribution of ITAG2.4 genes, expected *Pst*I cutting sites, *Pst*I cutting sites in the deep sequencing regions and RADseq SNPs, respectively. The black lines in the inner D layer indicate the heterochromatic regions.

The observation that 67.26% (16,365 to 24,330) of the SNPs were located in the annotated gene regions (Table 1) implied a correlation between the distribution of the identified SNPs in the current study and the distribution of the annotated genes. Additional observations in the current study indicated a preference for genomic DNA digestion by the *Pst*I restriction enzyme in the euchromatic regions: only 28.97% (23,988 to 82,814) of *Pst*I sites were found in the deep sequencing regions, and 83.55% (20,043 to 23,988) of the deep sequencing regions were located in the euchromatic region (Table S2). It is worth noting that the current RADseq protocol did produce low coverage of sequencing reads in some *Pst*I sites (with a read depth less than 10), and these *Pst*I sites were filtered by the criteria of SNP and genotype calling; therefore, the deep sequencing regions indicated that their read depths were no less than 10. Incidentally, because SNPs can be identified only in the sequenced regions, it is a reasonable deduction that most SNPs found in the current study are located in the euchromatic regions. Figure 1 confirms clearly that the annotated tomato genes (A layer), the *Pst*I sites in the deep sequencing regions (C layer), and identified SNPs (D layer) are mainly located in the euchromatic regions.

### Genetic differentiation of S. pimpinellifolium corresponded to the geographic area

**Figure 2:**
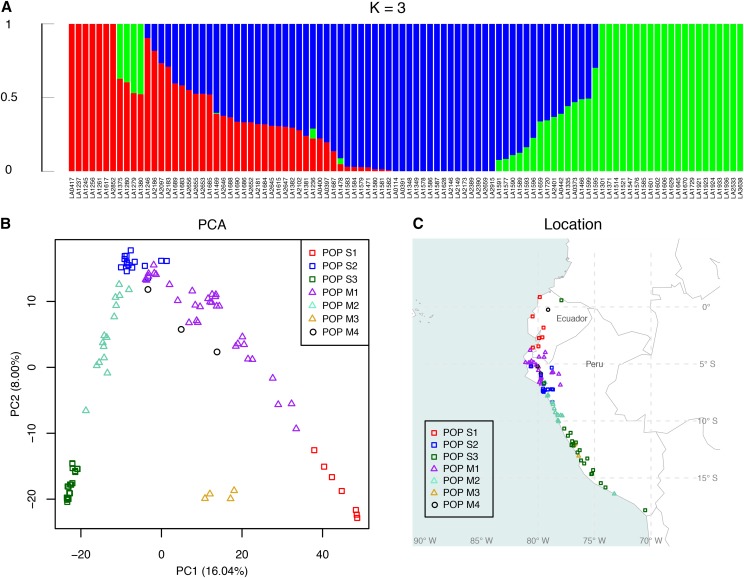
Ancestry and geographic distribution of 98 *Solanum pimpinellifolium* accessions from the Tomato Genetics Resource Center. A) Model-based ancestry for each accession. B) Principle component analysis of the *S. pimpinellifolium* population. C) Geographical distribution of the 98 *S. pimpinellifolium* accessions. Symbol and color codes are as follows: square symbols with red, blue and green colors indicate three single-ancestry subpopulations corresponding to the same colors in the ancestry plot (POP S1, POP S2 and POP S3, respectively); triangle symbols with purple, aquamarine and goldenrod colors present the POP M1, POP M2 and POP M3, respectively; black circle symbols were the POP M4.

The collection of 98 *S. pimpinellifolium* accessions was divided into three single-ancestry subpopulations and four mixed-ancestry subpopulations by the ADMIXTURE software (Figure 2A and Figure S1). We named the red, blue, and green single-ancestry subpopulations POP S1, POP S2, and POP S3, respectively (Table 2). Meanwhile, the red-blue, blue-green, red-green, and red-blue-green mixed-ancestry subpopulations were named as POP M1, POP M2, POP M3, and POP M4, respectively (Table 2). POP S1, POP S2, and POP S3 were clustered separately in the PCA plot, in which the first and the second principal components counted for 16.04% and 8.00% of the variance, respectively (Figure 2B). Moreover, pairwise *F_st_* confirmed the genetic differentiation (Table S4), and AMOVA revealed that the variance between subpopulations was 41.96% (p-value < 0.001).

**Table 2:** Genetic variation of each subpopulation

Subpopulation ID*^a^*	Genome pattern in ADMIXTURE	Sample size	Missing (%)	H_o_*^b^*	H_s_*^c^*
**Total**		**98**	**5.72**	**0.0761**	**0.2786**
POP S1	Red	7	6.14	0.0660	0.1856
POP S2	Blue	15	4.87	0.0558	0.1947
POP S3	Green	21	6.70	0.0451	0.1549
POP M1	Red-Blue	33	6.57	0.0948	0.2714
POP M2	Blue-Green	15	3.63	0.0779	0.1913
POP M3	Red-Green	4	4.78	0.1188	0.2133
POP M4	Red-Blue-Green	3	4.45	0.1468	0.1850

a : POP S indicates single ancestral subpopulation; POP M indicates mixed ancestral subpopulation.

b : H_o_ indicates the observed heterozygosity.

c : H_s_ indicates the within-population gene diversity (or “expected heterozygosity”).

The within-population gene diversity was calculated to compare genetic variation within each subpopulation. POP S2 and POP M1 showed the highest genetic variation among the single-ancestry subpopulations and the mixed-ancestry subpopulations, respectively (Table 2). Both subpopulations were in northern Peru, which indicated that northern Peru is the origin of *S. pimpinellifolium*.

Interestingly, most accessions in the same subpopulation were in the same vicinity of their collection sites (Figure 2C). Also, POP S1, POP S2, and POP S3 spread in somewhat distinct geographic areas along the coastline from Ecuador to southern Peru (Figure 2C). The geographic distribution of these subpopulations appeared in the following order from north to south: POP S1, POP M1, POP S2, POP M2, and POP S3 (Figure 2C). This geographic distribution showed a trend in which the mixed-ancestry subpopulations were located between their corresponding single-ancestry subpopulations.

### Rapid LD decay

**Figure 3:**
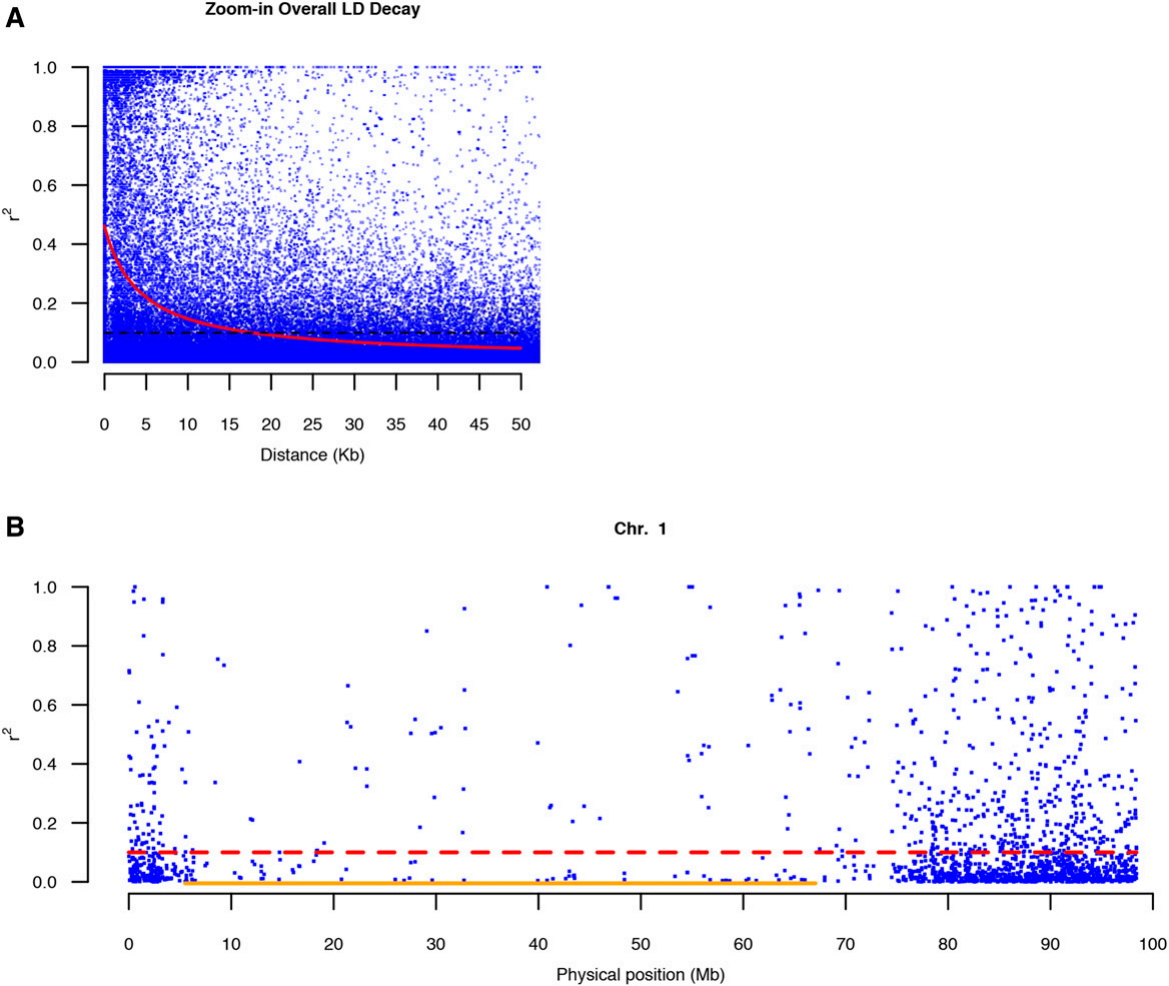
Visualization for LD. A) The 50 Kb interval of overall LD decay. The red curve indicates non-linear regression, and the black dotted line indicates the baseline of *r^2^* at 0.1. B) The local LD of chromosome 1. The red dotted line indicates the baseline of *r^2^*, and the orange line indicated the heterochromatic region.

LD decay was estimated for the mapping resolution in GWAS. In this population, the non-linear regression curve dropped very quickly (Figure S2). Following the non-linear regression curve, the overall LD decay was within 18 Kb when the baseline of the *r^2^* value was set at 0.1 (Table 3 and Figure 3A). The fastest LD decay was within 10 Kb on chromosome 9 while the slowest LD decay was within 30 Kb on chromosome 4 (Table 3 and Figure S3).

**Table 3:** The local LD profiles of individual chromosomes

Chr.	LD decay (Kb)	For paired flanking sequencing units		Proportion of LD for paired flanking sequencing units (%)
		Number of *r^2^* ≥ 0.1	Number of *r^2^* < 0.1	
1	14	632	1,130	35.87
2	12	475	881	35.03
3	15	460	927	33.17
4	30	423	687	38.11
5	21	309	514	37.55
6	20	428	750	36.66
7	21	397	581	40.59
8	28	401	618	39.35
9	10	280	617	31.22
10	19	330	525	38.60
11	19	310	535	36.69
12	17	253	539	31.94
**Total**	**18**	**4,698**	**8,304**	**36.13**

### Heterogeneity of genetic recombination within each chromosome

LD decay of individual chromosomes was insufficient to capture the local variations of historically accumulated recombination events because the tomato genome comprises more than 75% heterochromatin which usually suppresses recombination events (Sim *et al.* 2012a). We assessed the local LD profile of individual chromosomes based on the average *r^2^* value of flanking sequencing units that contained at least one SNP marker. We observed two main trends: marker density in the heterochromatic regions was lower than that in the euchromatic regions (Figure 3B and Figure S4), and approximately two-thirds of the *r^2^* values were less than 0.1 (Table 3). The latter observation indicated that these flanking SNP markers were not in a state of linkage disequilibrium.

## Discussion

### Subpopulations clustering from north to south are expected due to the high correlation between genetic distance and geographic distance

The genetic differentiation revealed in this study should be similar to previous findings because the collection sites of this collection cover most of recorded habitats of *S. pimpinellifolium*. One previous study for the genetic diversity of *S. pimpinellifolium* assessed 213 accessions with the genotypes of 10 SSR markers. It suggested the existence of Peruvian and Ecuadorian subpopulations (Zuriaga *et al.* 2009). Another study investigated a collection of 190 *S. pimpinellifolium* accessions using 48 SSR markers (Rao *et al.* 2012). It evaluated 120 accessions collected from Peru and 31 accessions from Ecuador, and divided these accessions into two single-ancestry subpopulations and one mixed-ancestry subpopulation. One of the single-ancestry subpopulation contained 93 accessions from Peru and 3 Ecuadorian accessions. These three Ecuadorian accessions were the only Ecuadorian accessions that were grouped into this single-ancestry subpopulation that contained mainly the Peruvian accessions, and the duplicated entries with the same names of these Ecuadorian accessions (LA0411, LA1246, LA1261) in the same study were grouped into the other two subpopulations. Despite of these three confounded Ecuadorian accessions, this study still inferred strong correlation between genetic diversity and geographic distance between Peruvian and Ecuadorian subpopulations (Rao *et al.* 2012). With the aid of SolCAP array, two consecutive studies, one with 63 *S. pimpinellifolium* accessions and the other with 112 *S. pimpinellifolium* accessions, sorted *S. pimpinellifolium* into three subpopulations: one in northern Ecuador, another in the mountainous area from southern Ecuador extending to northern Peru and the third in the low-altitude areas of Peru (Blanca *et al.* 2012, 2015). Our study also supports three single-ancestry subpopulations: one in Ecuador, one in northern Peru, and another in southern Peru. Among all the aforementioned studies, two ancestry subpopulations are confident: one includes the accessions in Ecuador; the other includes the accessions in southern Peru. The different grouping among these studies for those accessions from southern Ecuador to northern Peru may result from different markers and different genetic diversity in each study.

Previous studies suggested that genetic differentiation of *S. pimpinellifolium* correlated to the climatic variation (Rick *et al.* 1977; Zuriaga *et al.* 2009; Blanca *et al.* 2012, 2015). The analysis of genetic differentiation based on the RADseq data in the current study supported the same conclusion: most POP S1 accessions are in hot and humid Ecuador; most POP M1 scatter in northern Peru, along the western Andean slopes, in which is a warm desert; most POP S2 are located in the Andean Mountains; most POP M2 are in a warm semi-arid region; most POP S3 spread along the coastal region from central to southern Peru, in which is a relatively cold desert (Table S1 and Figure 2C). Since these subpopulations are located in the environments with different climates, and *F_st_* as well as AMOVA support these subpopulations (Table S4), the genetic differentiation of *S. pimpinellifolium* is observed evidently with the aid of RADseq SNP markers.

Isolation by distance (IBD) is a common tool to access genetic differentiation that expect a positive correlation between genetic variation and geographic distance (Wright 1943). We conducted this analysis for two datasets, the RADseq data and the SolCAP meta-data (File S1 and File S2), and made comparisons. The former data had the correlation coefficient equal to 0.34, and the latter one was 0.55 (Figure S5 and Figure S6). It seems that the RADseq data showed less genetic differentiation than the SolCAP meta-data. However, it has been argued that IBD test can be severely biased in two situations: unequal migration among all populations in a system, and the detection of loci under selection (Meirmans 2012). We do not know whether the investigated accessions were equally migrated, but we do know that the SolCAP array was designed mainly on the SNP sites of coding sequences within cultivated tomatoes or between cultivated tomato and wild tomatoes (Sim *et al.* 2012b). Therefore, the SNPs on the SolCAP array had higher chances under selection in domestication. Under this premise, the comparisons of the IBD test between the RADseq data and the SolCAP array data could be confounded by the differences in selection strength.

### Discrepancy of genetic clustering in SolCAP meta-analysis

Our meta-analysis concluded that the genetic compositions of 214 samples came from 15 ancestry populations. This conclusion is different from the conclusion of Blanca *et al.* (2012) and our RADseq data, both of which suggested that there were three ancestry populations of *S. pimpinellifolium*. This meta-analysis implied an unclear structure; especially the cross validation error has an ambiguous minimal value (Figure S7). It is possible that genetic diversity between wild tomatoes are underestimated because the polymorphisms of SolCAP array are selected between cultivars and wild tomatoes (Sim *et al.* 2012b). We noticed that two samples of LA0373 with 76% identity display different genome patterns in ADMIXTURE, while two samples of LA1478 with 71% identity present different patterns as well (Table S7 and Figure S8). Since two samples of the same accession demonstrate dissimilar genome patterns, the SolCAP may be less appropriate to quantize the population structure of *S. pimpinellifolium*, especially when more samples are involved. Also, for the same reason, we cannot validate the genetic differentiation in the SolCAP meta-analysis by *F_st_* or AMOVA nor achieve a stable estimation of genetic differentiation in a scenario of more accessions via the SolCAP meta-analysis.

### More markers are required to cover through the genome of S. pimpinellifolium

The observed and expected heterozygosity of this population were 0.0761 and 0.2786, respectively, slightly higher than those in previous researches (Blanca *et al.* 2012, 2015). Since *S. pimpinellifolium* was detected with up to a 40% outcrossing rate (Rick *et al.* 1977) and demonstrated high genetic variation, it is expected to cause rapid LD decay. In this study, LD decay was within 18 Kb throughout the genome, which was much shorter than cultivated tomatoes (Sim *et al.* 2012a; Bauchet *et al.* 2017). However, to put at least one SNP marker within each of 18 Kb intervals in this genome, the 900-Mb tomato genome would require at least 50,000 markers to fulfill QTL detection in GWAS. Therefore, acquiring many SNPs using different methods is essential to conduct a GWAS in the *S. pimpinellifolium* population. Here, we proposed three possible approaches to increase markers. One is to increase the sample size evenly for each subpopulation (Brachi *et al.* 2011). Since approximately 64% of alleles were rare in this population, the augmentation of the subpopulation size may adjust rare alleles to common alleles, potentially increasing the SNPs without extending coverage. One is to construct DNA libraries with a frequently cutting restriction enzyme. This approach can be simulated and optimized *in silico* to balance sequencing resource between sample sizes and sequencing coverage (Shirasawa *et al.* 2016). Another is exome sequencing, a selective genome sequencing technology that selects desired sequencing regions by the hybridization of designed probes (Kaur and Gaikwad 2017). Based on tomato genome sequence information, such as the gene model or EST database, one could design different sets of probes to limit sequencing regions (Ruggieri *et al.* 2017). Given the approximately 110 Mb total gene length in the ITAG2.4 gene model, the potential coverage could reach 12% and all target the gene region. This exome sequencing strategy may be able to increase SNPs without increasing population size.
